# Supplementary material for: Autophagy mitigates ethanol-induced mitochondrial dysfunction and oxidative stress in esophageal keratinocytes
Source: PLoS One. 2020 Sep 23;15(9):e0239625. doi: 10.1371/journal.pone.0239625 (PMC7510980; doi:10.1371/journal.pone.0239625)
Supplement: S1 Fig — (PDF) [file pone.0239625.s001.pdf]

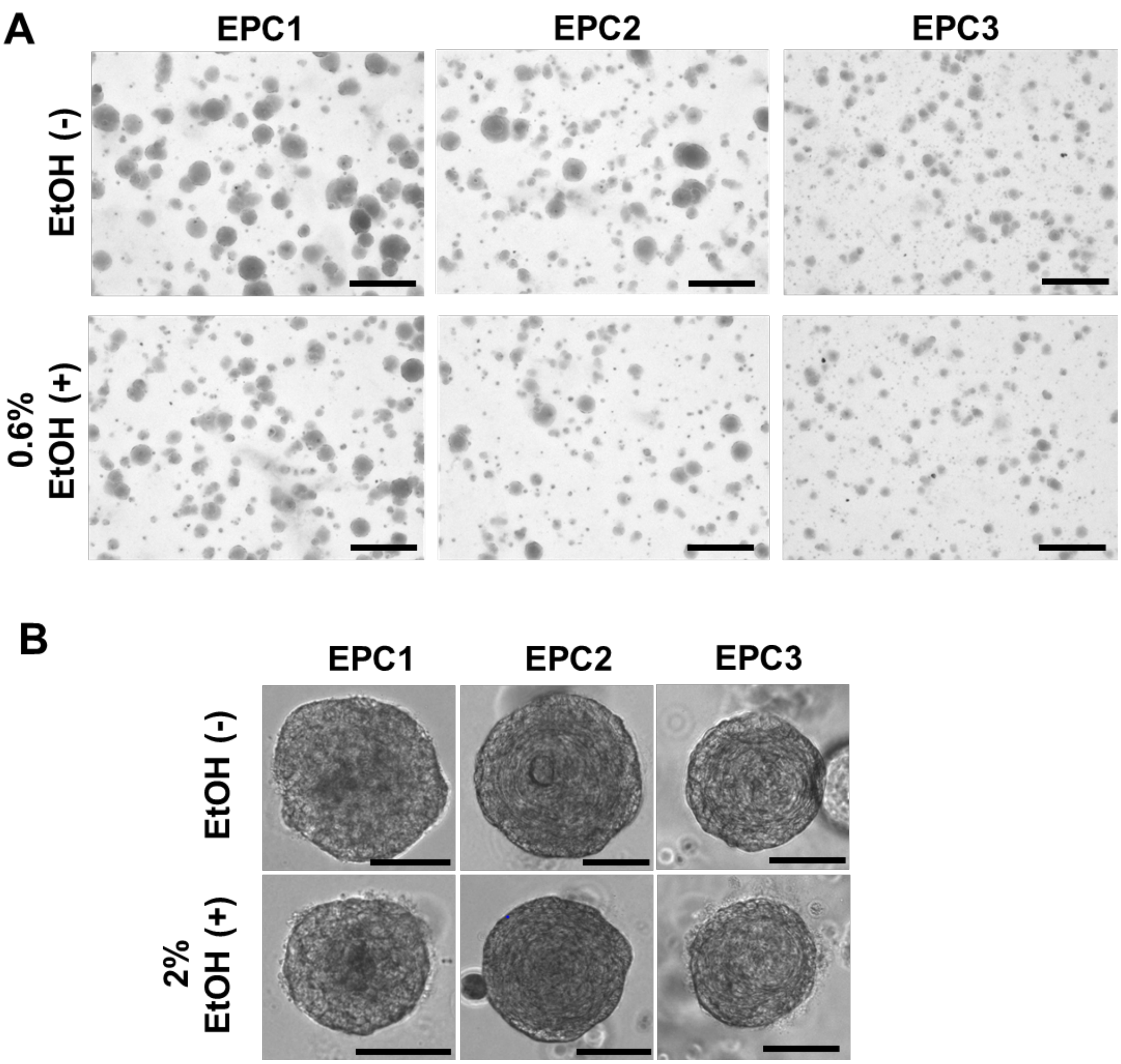

S1 Fig. Photomicrographs of human esophageal 3D organoids.

A. Representative bright-field images of 3D organoids growing in the presence or absence of 0.6% EtOH. Images were taken at day 10 in Fig 1A. Scale bars, 500 µm.

B. Representative phase-contrast images of 3D organoids untreated or treated with 2% EtOH in Fig 1B. Scale bars, 100 µm.
