# Supplementary material for: Autophagy mitigates ethanol-induced mitochondrial dysfunction and oxidative stress in esophageal keratinocytes
Source: PLoS One. 2020 Sep 23;15(9):e0239625. doi: 10.1371/journal.pone.0239625 (PMC7510980; doi:10.1371/journal.pone.0239625)
Supplement: S2 Fig — (PDF) [file pone.0239625.s002.pdf]

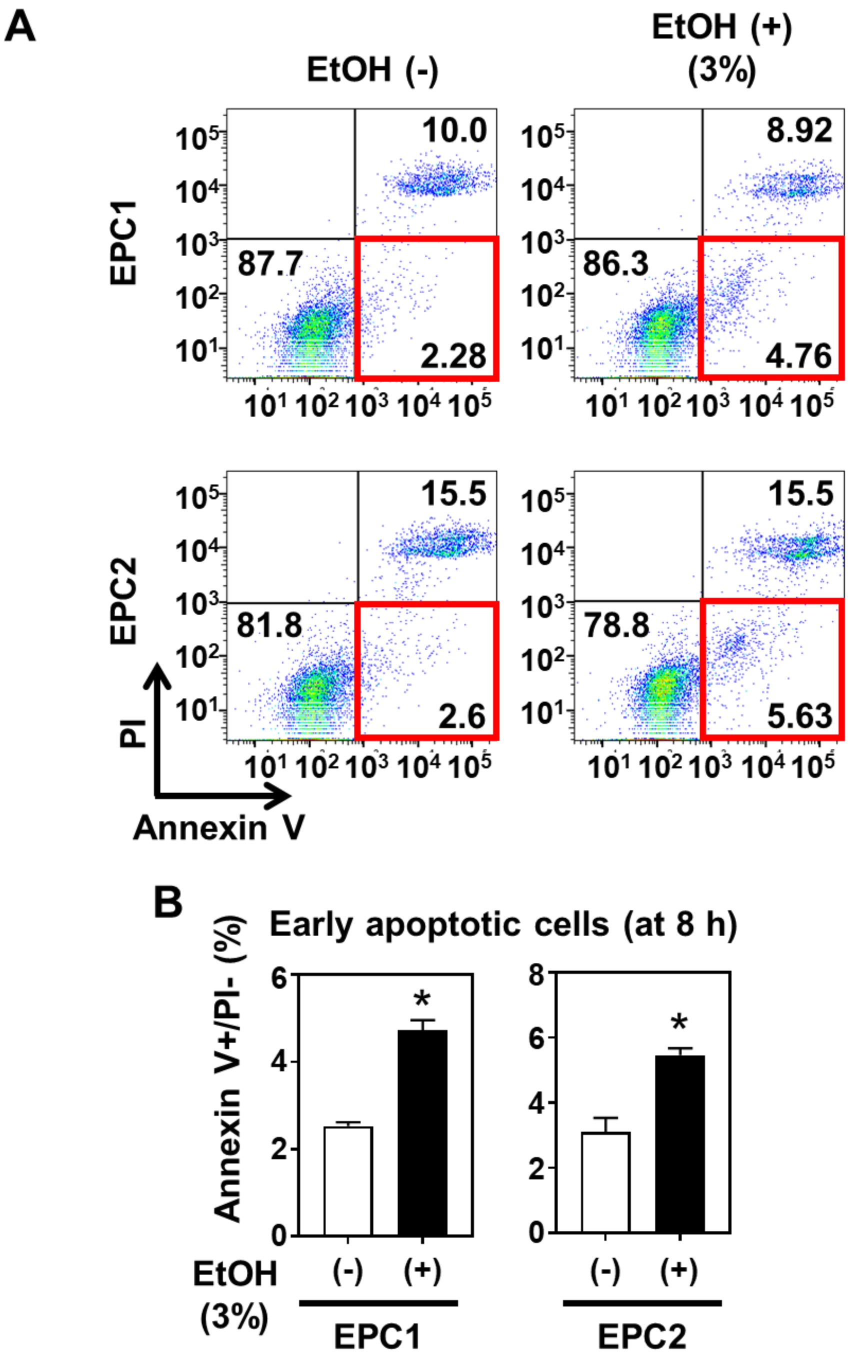

S2 Fig. **EtOH induces apoptosis in esophageal keratinocytes.**

EPC1 and EPC2 cells were treated with 3% EtOH for 8 h. Flow cytometry for Annexin-V and PI determined apoptosis with representative scatter plots (**A**) and bar diagram (mean ± sem, n=3 per condition) depicting average percentage of early apoptotic cells (Annexin-V-positive and PI-negative) shown in (**B**).

*, $p<0.05$ vs. EtOH (-), using student's t-test.
