# Supplementary material for: Autophagy mitigates ethanol-induced mitochondrial dysfunction and oxidative stress in esophageal keratinocytes
Source: PLoS One. 2020 Sep 23;15(9):e0239625. doi: 10.1371/journal.pone.0239625 (PMC7510980; doi:10.1371/journal.pone.0239625)
Supplement: S3 Fig — (PDF) [file pone.0239625.s003.pdf]

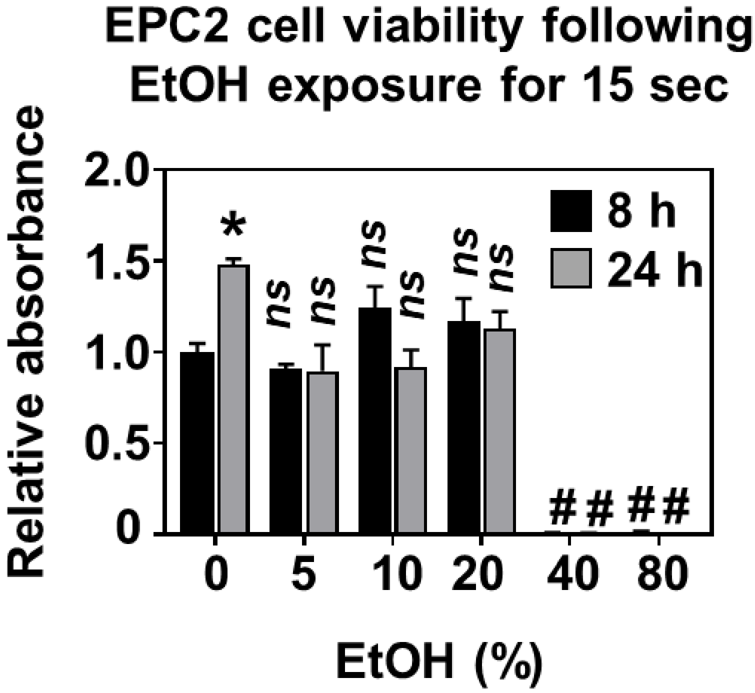

S3 Fig. **Effects of a short exposure to high concentrations of EtOH.**

EPC2 cells were grown in monolayer culture and exposed for 15 sec to PBS containing EtOH at indicated concentrations or PBS alone (control). Following replacement with cell culture medium to remove EtOH, cells were allowed to grow for up to 24 h and cell viability was determined by WST-1 assays at indicated time points. Data present mean ± sem. n=4 per condition. *, $p<0.05$ vs. 8 h and 0% EtOH; *ns*, not significant vs. 0% EtOH, either 8 h or 24 h; and #, $p<0.05$ vs. 0% EtOH, either 8 h or 24 h, using student's t-test.
