# Supplementary material for: Autophagy mitigates ethanol-induced mitochondrial dysfunction and oxidative stress in esophageal keratinocytes
Source: PLoS One. 2020 Sep 23;15(9):e0239625. doi: 10.1371/journal.pone.0239625 (PMC7510980; doi:10.1371/journal.pone.0239625)
Supplement: S4 Fig — (PDF) [file pone.0239625.s004.pdf]

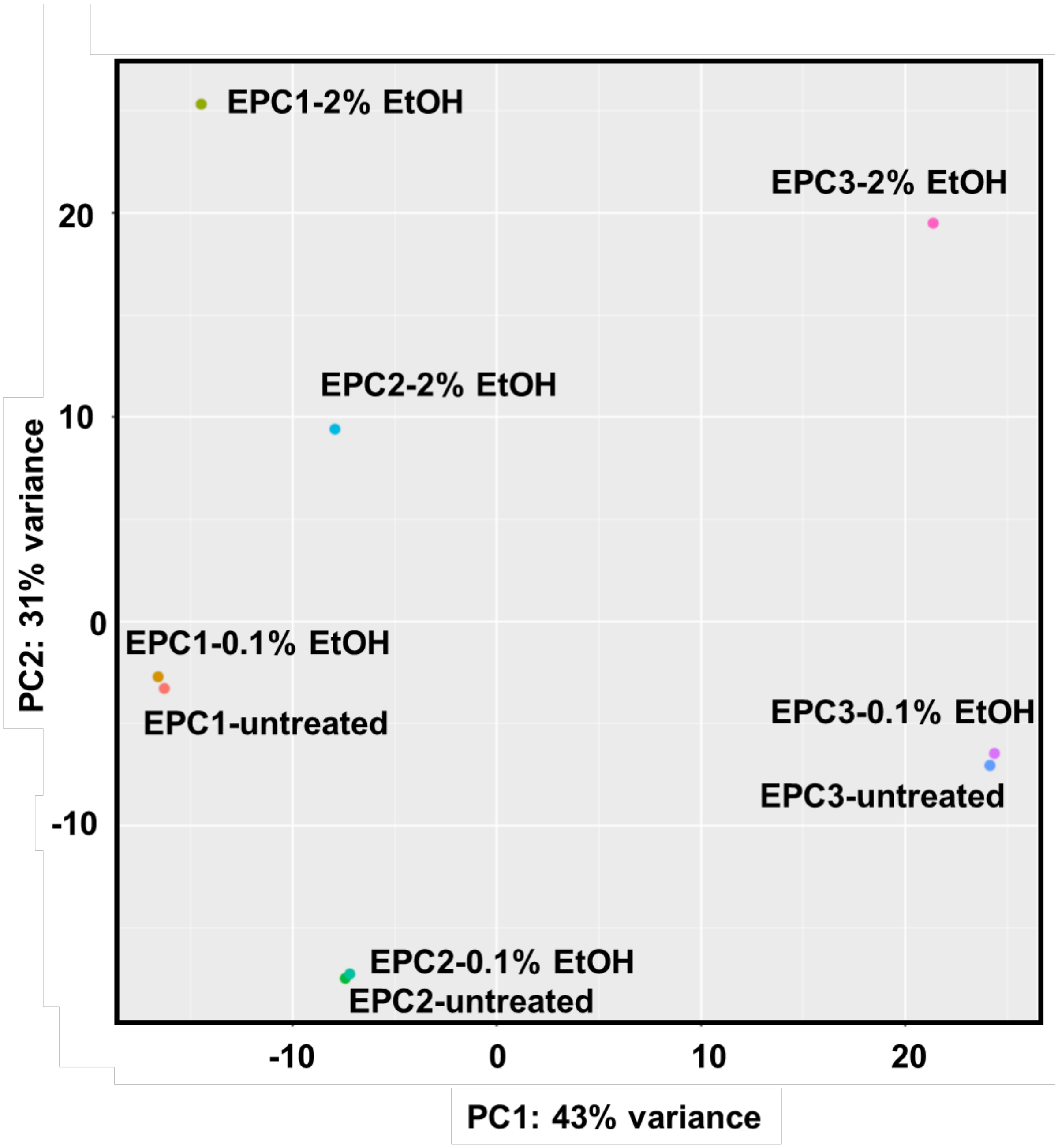

S4 Fig. Principal Component Analysis.

RNA-seq data from EPC1, EPC2 and EPC3 cells untreated or treated with 0.1% or 2% EtOH for 8 h were subjected to principal component analysis. Note that 0.1% EtOH had a minimal impact upon gene expression in all cell lines. EPC1 and EPC2 clustered closer than EPC3 under each treatment condition.
