# Supplementary material for: Autophagy mitigates ethanol-induced mitochondrial dysfunction and oxidative stress in esophageal keratinocytes
Source: PLoS One. 2020 Sep 23;15(9):e0239625. doi: 10.1371/journal.pone.0239625 (PMC7510980; doi:10.1371/journal.pone.0239625)
Supplement: S5 Fig — (PDF) [file pone.0239625.s005.pdf]

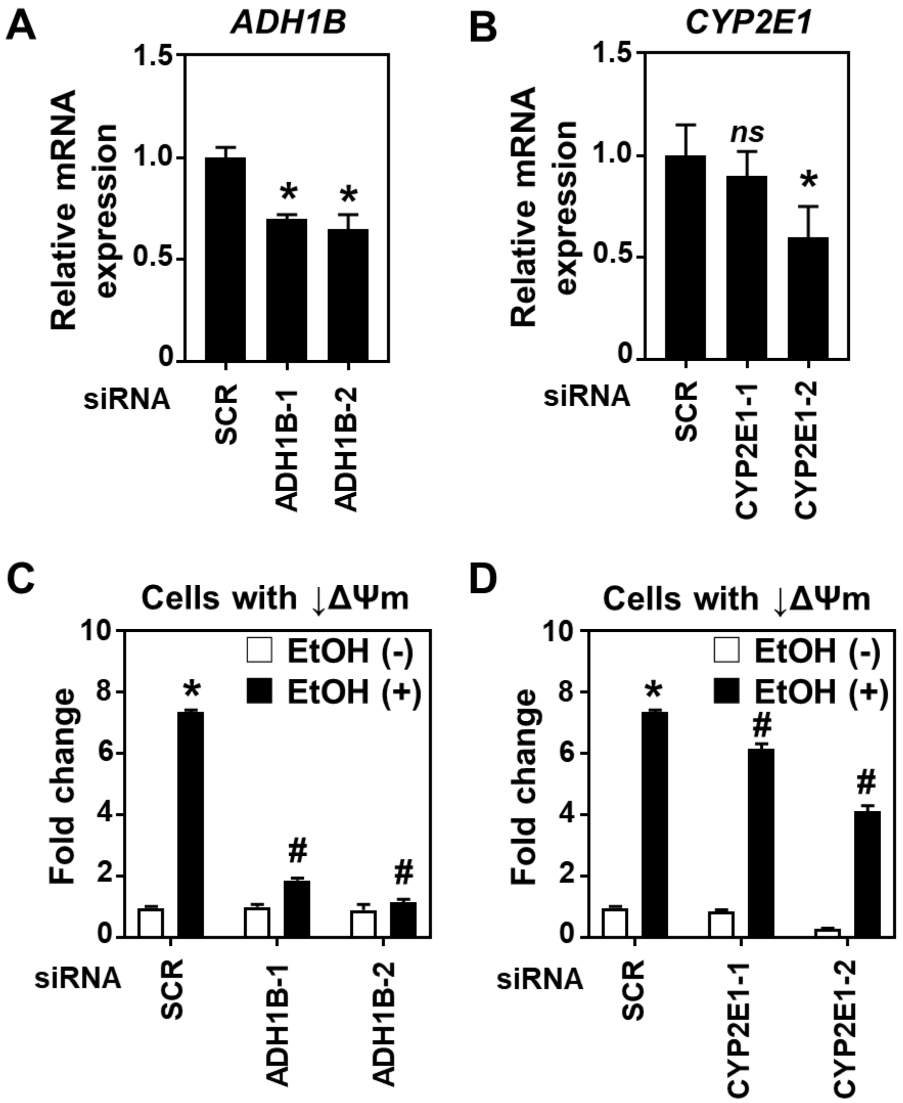

S5 Fig. ADH1B and CYP2E1 may mediate EtOH-induced mitochondria depolarization.

EPC2 cells were transfected with two independent siRNA sequences directed against *ADH1B*, *CYP2E1* or non-silencing scramble control RNA (SCR) 48 h before EtOH exposure. **A** and **B**. Quantitative RT-PCR assays were performed at 48 h following siRNA transfection to document the RNAi effect upon mRNA expression of *ADH1B* in **A** and *CYP2E1* in **B**. Bar diagrams show fold-difference in mRNA expression compared to SCR as 1. Data represent mean ± sem. n=3 per condition. *, p<0.05 vs. SCR; ns, not significant vs. SCR, using student's t-test.

C and D. Following siRNA transfection, cells were treated with or without 2% EtOH for 8h, and stained with MTG and MTDR for flow cytometry. Bar diagrams demonstrate quantitation (mean ± SEM) of the cells with decreased membrane potential (↓ΔΨm) expressed as a fold-change compared to SCR and EtOH (-) as 1. n=3 per condition. *p<0.05 vs. SCR and EtOH (-), #p<0.05 vs. SCR and EtOH (+), using student's t-test.
