# Supplementary material for: Autophagy mitigates ethanol-induced mitochondrial dysfunction and oxidative stress in esophageal keratinocytes
Source: PLoS One. 2020 Sep 23;15(9):e0239625. doi: 10.1371/journal.pone.0239625 (PMC7510980; doi:10.1371/journal.pone.0239625)
Supplement: S6 Fig — (PDF) [file pone.0239625.s006.pdf]

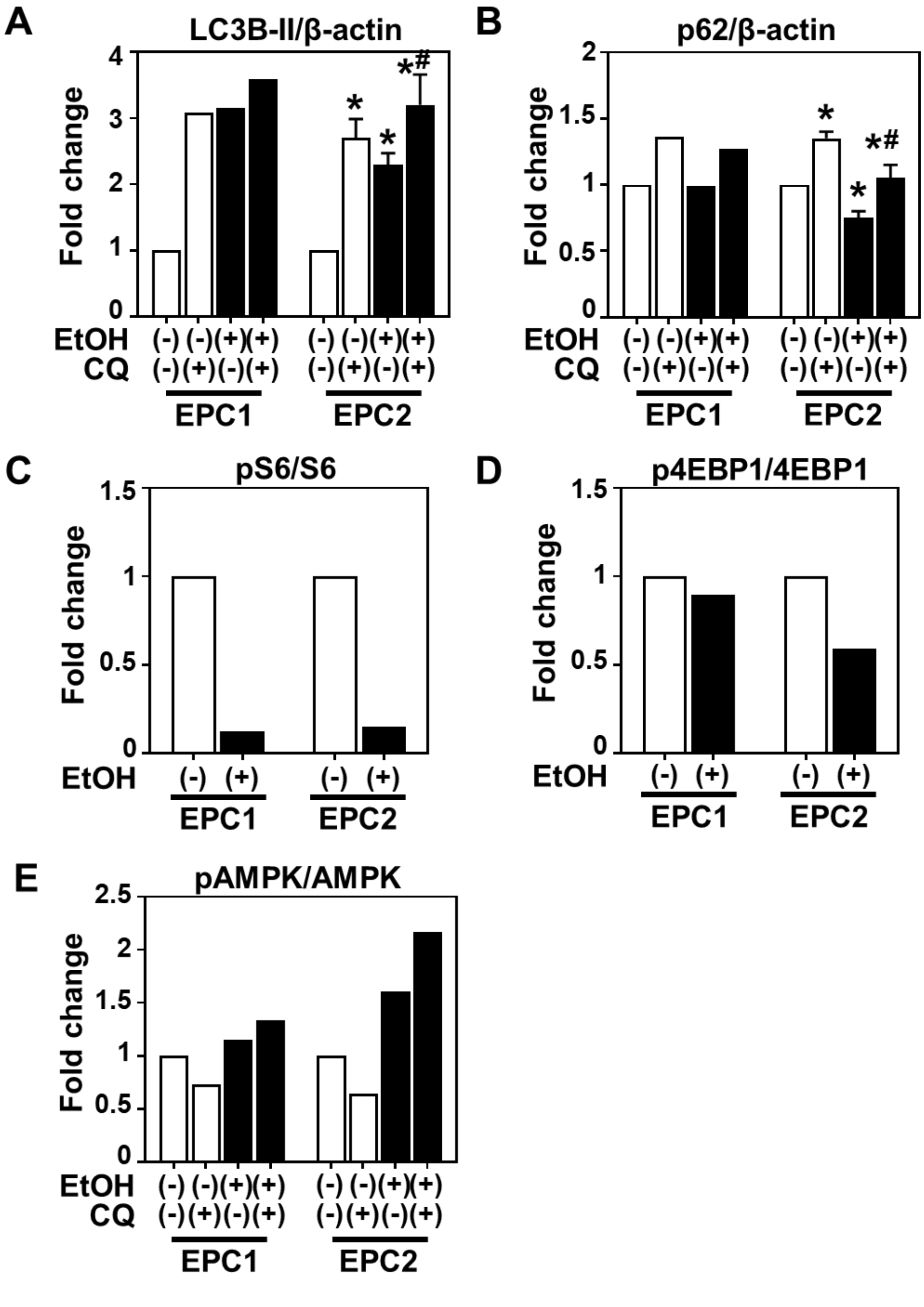

S6 Fig. Densitometry of immunoblots.

Bar diagrams show relative signal intensity of representative immunoblots in Fig 7A, Fig 9C and Fig 10A. Statistical analysis was done for EPC2 data only (mean ± sem, n=3 per condition) in **A** (LC3-II) and **B** (p62) where three immunoblots (i.e. three independent experiments done on different days) were subjected to densitometry. *, $p<0.05$ vs. EtOH (-) CQ (-); #, $p<0.05$ vs. EtOH (+) CQ (-), using student's t-test.
