# Supplementary material for: Autophagy mitigates ethanol-induced mitochondrial dysfunction and oxidative stress in esophageal keratinocytes
Source: PLoS One. 2020 Sep 23;15(9):e0239625. doi: 10.1371/journal.pone.0239625 (PMC7510980; doi:10.1371/journal.pone.0239625)
Supplement: S1 Table — (PDF) [file pone.0239625.s010.pdf]

S1 Table RNA-seq Reads Summary

Sample	Input Reads	Uniquely Mapped Reads
EPC1_0% EtOH	25,272,596	21,577,936
EPC1_0.1% EtOH	29,687,837	25,676,964
EPC1_2% EtOH	29,308,671	248,770,20
EPC2_0% EtOH	31,972,728	28,046,906
EPC2_0.1% EtOH	45,660,092	38,480,547
EPC2_2% EtOH	33,600,923	28,466,139
EPC3_0% EtOH	29,717,883	14,071,151
EPC3_0.1% EtOH	30,979,461	14,624,460
EPC3_2% EtOH	31,606,802	19,250,686
AVG	31,978,555	23,896,868
