# Supplementary material for: Autophagy mitigates ethanol-induced mitochondrial dysfunction and oxidative stress in esophageal keratinocytes
Source: PLoS One. 2020 Sep 23;15(9):e0239625. doi: 10.1371/journal.pone.0239625 (PMC7510980; doi:10.1371/journal.pone.0239625)
Supplement: S2 Table — (PDF) [file pone.0239625.s011.pdf]

S2 Table. Complete list of genes related to mitochondrial biogenesis and functions that were identified by RNA-seq to be significantly negatively enriched in the context of alcohol-induced stress in esophageal keratinocytes. (NES: Normalized enrichment score, FDR: False discovery rate)

**ID: GO:0005743; Name: mitochondrial inner membrane**				
**Size=184; L=126; ES=-0.36; NES=-2.37; PValue=0.000e+0; FDR=8.337e-4**				
**User ID**	**Gene Symbol**	**Gene Name**	**Entrez Gene**	**Score**
ABCB10	ABCB10	ATP binding cassette subfamily B member 10	23456	-0.826
ABCB8	ABCB8	ATP binding cassette subfamily B member 8	11194	-0.6728
ACAD9	ACAD9	acyl-CoA dehydrogenase family member 9	28976	-1.494
AFG3L2	AFG3L2	AFG3 like matrix AAA peptidase subunit 2	10939	-0.4887
ALDH18A1	ALDH18A1	aldehyde dehydrogenase 18 family member A1	5832	-0.5769
ATAD3A	ATAD3A	ATPase family, AAA domain containing 3A	55210	-1.9207
ATAD3B	ATAD3B	ATPase family, AAA domain containing 3B	83858	-1.4723
ATP5F1A	ATP5F1A	ATP synthase F1 subunit alpha	498	-0.4815
ATP5F1D	ATP5F1D	ATP synthase F1 subunit delta	513	-0.7817
ATP5MC1	ATP5MC1	ATP synthase membrane subunit c locus 1	516	-0.8153
AURKAIP1	AURKAIP1	aurora kinase A interacting protein 1	54998	-0.7599
BCS1L	BCS1L	BCS1 homolog, ubiquinol-cytochrome c reductase complex chaperone	617	-0.8502
BDH1	BDH1	3-hydroxybutyrate dehydrogenase 1	622	-2.1539
BOK	BOK	BCL2 family apoptosis regulator BOK	666	-1.2302
CHCHD6	CHCHD6	coiled-coil-helix-coiled-coil-helix domain containing 6	84303	-0.5252
COA3	COA3	cytochrome c oxidase assembly factor 3	28958	-1.6093
COQ4	COQ4	coenzyme Q4	51117	-0.5793
COQ7	COQ7	coenzyme Q7, hydroxylase	10229	-0.6094
COQ9	COQ9	coenzyme Q9	57017	-0.5372
COX15	COX15	cytochrome c oxidase assembly homolog COX15	1355	-0.71
COX18	COX18	cytochrome c oxidase assembly factor COX18	285521	-1.1092
CYC1	CYC1	cytochrome c1	1537	-0.8942
CYCS	CYCS	cytochrome c, somatic	54205	-0.8343
DHFR2	DHFR2	dihydrofolate reductase 2	200895	-1.5204
DHODH	DHODH	dihydroorotate dehydrogenase (quinone)	1723	-1.7029
DMAC1	DMAC1	distal membrane arm assembly complex 1	90871	-0.7157
DMAC2	DMAC2	distal membrane arm assembly complex 2	55101	-0.8339
DMAC2L	DMAC2L	distal membrane arm assembly complex 2 like	27109	-0.8514
DNAJC11	DNAJC11	DnaJ heat shock protein family (Hsp40) member C11	55735	-0.9315
DNAJC30	DNAJC30	DnaJ heat shock protein family (Hsp40) member C30	84277	-1.8841
FOXRED1	FOXRED1	FAD dependent oxidoreductase domain containing 1	55572	-0.8658
FPGS	FPGS	folylpolyglutamate synthase	2356	-1.0053
GPD2	GPD2	glycerol-3-phosphate dehydrogenase 2	2820	-1.2221
GRPEL1	GRPEL1	GrpE like 1, mitochondrial	80273	-0.5003
IMMT	IMMT	inner membrane mitochondrial protein	10989	-0.4827
LETM1	LETM1	leucine zipper and EF-hand containing transmembrane protein 1	3954	-1.1449
MCU	MCU	mitochondrial calcium uniporter	90550	-0.6009
MICU2	MICU2	mitochondrial calcium uptake 2	221154	-0.8139
MPV17	MPV17	mitochondrial inner membrane protein MPV17	4358	-0.5302
MRPL1	MRPL1	mitochondrial ribosomal protein L1	65008	-0.8841
MRPL12	MRPL12	mitochondrial ribosomal protein L12	6182	-1.8151
MRPL15	MRPL15	mitochondrial ribosomal protein L15	29088	-0.9279
MRPL16	MRPL16	mitochondrial ribosomal protein L16	54948	-0.7676
MRPL17	MRPL17	mitochondrial ribosomal protein L17	63875	-1.2273
MRPL19	MRPL19	mitochondrial ribosomal protein L19	9801	-0.6191
MRPL2	MRPL2	mitochondrial ribosomal protein L2	51069	-0.6308
MRPL20	MRPL20	mitochondrial ribosomal protein L20	55052	-0.9108
MRPL21	MRPL21	mitochondrial ribosomal protein L21	219927	-0.7527
MRPL24	MRPL24	mitochondrial ribosomal protein L24	79590	-0.7585
MRPL27	MRPL27	mitochondrial ribosomal protein L27	51264	-1.0032
MRPL28	MRPL28	mitochondrial ribosomal protein L28	10573	-0.9266
MRPL3	MRPL3	mitochondrial ribosomal protein L3	11222	-0.5017
MRPL32	MRPL32	mitochondrial ribosomal protein L32	64983	-0.65
MRPL35	MRPL35	mitochondrial ribosomal protein L35	51318	-1.0054
MRPL36	MRPL36	mitochondrial ribosomal protein L36	64979	-0.9912
MRPL37	MRPL37	mitochondrial ribosomal protein L37	51253	-0.5457
MRPL4	MRPL4	mitochondrial ribosomal protein L4	51073	-0.6947
MRPL42	MRPL42	mitochondrial ribosomal protein L42	28977	-0.5884
MRPL50	MRPL50	mitochondrial ribosomal protein L50	54534	-0.7727
MRPL52	MRPL52	mitochondrial ribosomal protein L52	122704	-0.8534

MRPL54	MRPL54	mitochondrial ribosomal protein L54	116541	-0.8803
MRPL55	MRPL55	mitochondrial ribosomal protein L55	128308	-0.5606
MRPL57	MRPL57	mitochondrial ribosomal protein L57	78988	-1.1413
MRPL58	MRPL58	mitochondrial ribosomal protein L58	3396	-1.0237
MRPL9	MRPL9	mitochondrial ribosomal protein L9	65005	-0.4901
MRPS12	MRPS12	mitochondrial ribosomal protein S12	6183	-1.0623
MRPS14	MRPS14	mitochondrial ribosomal protein S14	63931	-0.8098
MRPS16	MRPS16	mitochondrial ribosomal protein S16	51021	-0.5475
MRPS17	MRPS17	mitochondrial ribosomal protein S17	51373	-1.2133
MRPS18B	MRPS18B	mitochondrial ribosomal protein S18B	28973	-0.7077
MRPS2	MRPS2	mitochondrial ribosomal protein S2	51116	-0.7504
MRPS26	MRPS26	mitochondrial ribosomal protein S26	64949	-0.8028
MRPS27	MRPS27	mitochondrial ribosomal protein S27	23107	-0.6219
MRPS28	MRPS28	mitochondrial ribosomal protein S28	28957	-1.2515
MRPS30	MRPS30	mitochondrial ribosomal protein S30	10884	-0.6256
MRPS34	MRPS34	mitochondrial ribosomal protein S34	65993	-0.8679
MRPS35	MRPS35	mitochondrial ribosomal protein S35	60488	-0.4987
MRPS7	MRPS7	mitochondrial ribosomal protein S7	51081	-1.0273
MRPS9	MRPS9	mitochondrial ribosomal protein S9	64965	-0.4876
NDUFAF3	NDUFAF3	NADH:ubiquinone oxidoreductase complex assembly factor 3	25915	-0.924
NDUFAF4	NDUFAF4	NADH:ubiquinone oxidoreductase complex assembly factor 4	29078	-0.5502
NDUFS1	NDUFS1	NADH:ubiquinone oxidoreductase core subunit S1	4719	-0.7155
NDUFS2	NDUFS2	NADH:ubiquinone oxidoreductase core subunit S2	4720	-0.7935
NDUFS8	NDUFS8	NADH:ubiquinone oxidoreductase core subunit S8	4728	-0.5743
NDUFV1	NDUFV1	NADH:ubiquinone oxidoreductase core subunit V1	4723	-0.502
NDUFV3	NDUFV3	NADH:ubiquinone oxidoreductase subunit V3	4731	-1.2657
OPA1	OPA1	OPA1, mitochondrial dynamin like GTPase	4976	-0.5814
OXA1L	OXA1L	OXA1L, mitochondrial inner membrane protein	5018	-0.5463
PARL	PARL	presenilin associated rhomboid like	55486	-0.5397
PHB	PHB	prohibitin	5245	-0.4919
PHB2	PHB2	prohibitin 2	11331	-0.5923
PISD	PISD	phosphatidylserine decarboxylase	23761	-0.6885
PLA2G4A	PLA2G4A	phospholipase A2 group IVA	5321	-1.0082
PMPCA	PMPCA	peptidase, mitochondrial processing alpha subunit	23203	-1.3778
PTCD3	PTCD3	pentatricopeptide repeat domain 3	55037	-0.7189
SDHA	SDHA	succinate dehydrogenase complex flavoprotein subunit A	6389	-1.2335
SDHAF4	SDHAF4	succinate dehydrogenase complex assembly factor 4	135154	-0.9228
SFXN4	SFXN4	sideroflexin 4	119559	-0.9558
SLC25A1	SLC25A1	solute carrier family 25 member 1	6576	-0.7313
SLC25A10	SLC25A10	solute carrier family 25 member 10	1468	-1.5629
SLC25A11	SLC25A11	solute carrier family 25 member 11	8402	-1.3604
SLC25A13	SLC25A13	solute carrier family 25 member 13	10165	-1.1533
SLC25A15	SLC25A15	solute carrier family 25 member 15	10166	-1.0959
SLC25A19	SLC25A19	solute carrier family 25 member 19	60386	-0.8701
SLC25A20	SLC25A20	solute carrier family 25 member 20	788	-1.1446
SLC25A22	SLC25A22	solute carrier family 25 member 22	79751	-0.5821
SLC25A37	SLC25A37	solute carrier family 25 member 37	51312	-0.6514
SPG7	SPG7	SPG7, paraplegin matrix AAA peptidase subunit	6687	-0.709
SQOR	SQOR	sulfide quinone oxidoreductase	58472	-0.624
STOML2	STOML2	stomatin like 2	30968	-0.6088
TIMM10B	TIMM10B	translocase of inner mitochondrial membrane 10B	26515	-1.1482
TIMM13	TIMM13	translocase of inner mitochondrial membrane 13	26517	-0.8215
TIMM17A	TIMM17A	translocase of inner mitochondrial membrane 17A	10440	-0.6497
TIMM21	TIMM21	translocase of inner mitochondrial membrane 21	29090	-0.5245
TIMM22	TIMM22	translocase of inner mitochondrial membrane 22	29928	-0.6646
TIMM50	TIMM50	translocase of inner mitochondrial membrane 50	92609	-0.5457
TIMM8A	TIMM8A	translocase of inner mitochondrial membrane 8A	1678	-1.4931
TIMM8B	TIMM8B	translocase of inner mitochondrial membrane 8 homolog B	26521	-0.6737
TMEM126B	TMEM126B	transmembrane protein 126B	55863	-0.5568
TMEM177	TMEM177	transmembrane protein 177	80775	-1.0773
TOMM40	TOMM40	translocase of outer mitochondrial membrane 40	10452	-1.6586
TRAP1	TRAP1	TNF receptor associated protein 1	10131	-0.7914
TTC19	TTC19	tetratricopeptide repeat domain 19	54902	-0.6218
TYMS	TYMS	thymidylate synthetase	7298	-1.1017
UQCC2	UQCC2	ubiquinol-cytochrome c reductase complex assembly factor 2	84300	-1.0467
UQCRC2	UQCRC2	ubiquinol-cytochrome c reductase core protein 2	7385	-0.6507

**ID: GO:0005759; Name: mitochondrial matrix**				
**Size=219; L=135; ES=-0.35; NES=-2.34; PValue=0.000e+0; FDR=6.669e-4**				
**User ID**	**Gene Symbol**	**Gene Name**	**Entrez Gene**	**Score**
AADAT	AADAT	aminoadipate aminotransferase	51166	-0.6175
ABCE1	ABCE1	ATP binding cassette subfamily E member 1	6059	-1.4977
ACAD10	ACAD10	acyl-CoA dehydrogenase family member 10	80724	-0.7077
ACAT1	ACAT1	acetyl-CoA acetyltransferase 1	38	-0.6097
ACO2	ACO2	aconitase 2	50	-0.5616
ACSF3	ACSF3	acyl-CoA synthetase family member 3	197322	-0.6583
ADPRHL2	ADPRHL2	ADP-ribosylhydrolase like 2	54936	-0.7834
AK4	AK4	adenylate kinase 4	205	-0.6986
ALDH1B1	ALDH1B1	aldehyde dehydrogenase 1 family member B1	219	-0.9382
ATAD3A	ATAD3A	ATPase family, AAA domain containing 3A	55210	-1.9207
ATP5F1D	ATP5F1D	ATP synthase F1 subunit delta	513	-0.7817
BCKDHB	BCKDHB	branched chain keto acid dehydrogenase E1 subunit beta	594	-0.8234
BCKDK	BCKDK	branched chain ketoacid dehydrogenase kinase	10295	-1.1649
BDH1	BDH1	3-hydroxybutyrate dehydrogenase 1	622	-2.1539
BPHL	BPHL	biphenyl hydrolase like	670	-0.8877
C1QBP	C1QBP	complement C1q binding protein	708	-0.8139
CCAR2	CCAR2	cell cycle and apoptosis regulator 2	57805	-1.0632
CDK1	CDK1	cyclin dependent kinase 1	983	-0.7493
CLPP	CLPP	caseinolytic mitochondrial matrix peptidase proteolytic subunit	8192	-0.5769
CREB1	CREB1	cAMP responsive element binding protein 1	1385	-1.6345
D2HGDH	D2HGDH	D-2-hydroxyglutarate dehydrogenase	728294	-0.9099
DARS2	DARS2	aspartyl-tRNA synthetase 2, mitochondrial	55157	-0.6151
DDX28	DDX28	DEAD-box helicase 28	55794	-0.994
DHFR2	DHFR2	dihydrofolate reductase 2	200895	-1.5204
DHTKD1	DHTKD1	dehydrogenase E1 and transketolase domain containing 1	55526	-0.7223
DHX30	DHX30	DExH-box helicase 30	22907	-0.7761
DIMT1	DIMT1	DIM1 dimethyladenosine transferase 1 homolog	27292	-1.2703
DNA2	DNA2	DNA replication helicase/nuclease 2	1763	-1.2112
DNAJA3	DNAJA3	DnaJ heat shock protein family (Hsp40) member A3	9093	-0.798
DTYMK	DTYMK	deoxythymidylate kinase	1841	-0.8633
EARS2	EARS2	glutamyl-tRNA synthetase 2, mitochondrial	124454	-1.3235
ELAC2	ELAC2	elaC ribonuclease Z 2	60528	-1.178
ETFBKMT	ETFBKMT	electron transfer flavoprotein subunit beta lysine methyltransferase	254013	-0.8796
FAHD1	FAHD1	fumarylacetoacetate hydrolase domain containing 1	81889	-1.891
FARS2	FARS2	phenylalanyl-tRNA synthetase 2, mitochondrial	10667	-0.7725
FASTK	FASTK	Fas activated serine/threonine kinase	10922	-0.7087
FASTKD2	FASTKD2	FAST kinase domains 2	22868	-0.8108
FH	FH	fumarate hydratase	2271	-0.8757
FLAD1	FLAD1	flavin adenine dinucleotide synthetase 1	80308	-0.7522
FPGS	FPGS	folylpolyglutamate synthase	2356	-1.0053
FXN	FXN	frataxin	2395	-1.1031
GADD45GIP1	GADD45GIP1	GADD45G interacting protein 1	90480	-0.9754
GCDH	GCDH	glutaryl-CoA dehydrogenase	2639	-1.6761
GFM1	GFM1	G elongation factor mitochondrial 1	85476	-0.9184
GPX1	GPX1	glutathione peroxidase 1	2876	-0.6268
HADH	HADH	hydroxyacyl-CoA dehydrogenase	3033	-0.6742
HSPA9	HSPA9	heat shock protein family A (Hsp70) member 9	3313	-0.874
IDH3A	IDH3A	isocitrate dehydrogenase 3 (NAD(+)) alpha	3419	-0.8748
IDH3G	IDH3G	isocitrate dehydrogenase 3 (NAD(+)) gamma	3421	-0.9346
IVD	IVD	isovaleryl-CoA dehydrogenase	3712	-0.7884
KARS	KARS	lysyl-tRNA synthetase	3735	-0.6239
LACTB2	LACTB2	lactamase beta 2	51110	-1.1198
LRRC59	LRRC59	leucine rich repeat containing 59	55379	-0.6982
LYRM7	LYRM7	LYR motif containing 7	90624	-1.389
MARS2	MARS2	methionyl-tRNA synthetase 2, mitochondrial	92935	-0.9665
MCAT	MCAT	malonyl-CoA-acyl carrier protein transacylase	27349	-0.964
MECR	MECR	mitochondrial trans-2-enoyl-CoA reductase	51102	-1.2
MIPEP	MIPEP	mitochondrial intermediate peptidase	4285	-1.2205
MMAB	MMAB	metabolism of cobalamin associated B	326625	-0.5986
MPG	MPG	N-methylpurine DNA glycosylase	4350	-0.7103
MRPL12	MRPL12	mitochondrial ribosomal protein L12	6182	-1.8151
MRPL15	MRPL15	mitochondrial ribosomal protein L15	29088	-0.9279
MRPL16	MRPL16	mitochondrial ribosomal protein L16	54948	-0.7676

MRPL17	MRPL17	mitochondrial ribosomal protein L17	63875	-1.2273
MRPL19	MRPL19	mitochondrial ribosomal protein L19	9801	-0.6191
MRPL2	MRPL2	mitochondrial ribosomal protein L2	51069	-0.6308
MRPL20	MRPL20	mitochondrial ribosomal protein L20	55052	-0.9108
MRPL21	MRPL21	mitochondrial ribosomal protein L21	219927	-0.7527
MRPL24	MRPL24	mitochondrial ribosomal protein L24	79590	-0.7585
MRPL27	MRPL27	mitochondrial ribosomal protein L27	51264	-1.0032
MRPL28	MRPL28	mitochondrial ribosomal protein L28	10573	-0.9266
MRPL32	MRPL32	mitochondrial ribosomal protein L32	64983	-0.65
MRPL35	MRPL35	mitochondrial ribosomal protein L35	51318	-1.0054
MRPL36	MRPL36	mitochondrial ribosomal protein L36	64979	-0.9912
MRPL37	MRPL37	mitochondrial ribosomal protein L37	51253	-0.5457
MRPL4	MRPL4	mitochondrial ribosomal protein L4	51073	-0.6947
MRPL42	MRPL42	mitochondrial ribosomal protein L42	28977	-0.5884
MRPL50	MRPL50	mitochondrial ribosomal protein L50	54534	-0.7727
MRPL52	MRPL52	mitochondrial ribosomal protein L52	122704	-0.8534
MRPL54	MRPL54	mitochondrial ribosomal protein L54	116541	-0.8803
MRPL55	MRPL55	mitochondrial ribosomal protein L55	128308	-0.5606
MRPL57	MRPL57	mitochondrial ribosomal protein L57	78988	-1.1413
MRPL58	MRPL58	mitochondrial ribosomal protein L58	3396	-1.0237
MRPS12	MRPS12	mitochondrial ribosomal protein S12	6183	-1.0623
MRPS14	MRPS14	mitochondrial ribosomal protein S14	63931	-0.8098
MRPS16	MRPS16	mitochondrial ribosomal protein S16	51021	-0.5475
MRPS17	MRPS17	mitochondrial ribosomal protein S17	51373	-1.2133
MRPS18B	MRPS18B	mitochondrial ribosomal protein S18B	28973	-0.7077
MRPS2	MRPS2	mitochondrial ribosomal protein S2	51116	-0.7504
MRPS26	MRPS26	mitochondrial ribosomal protein S26	64949	-0.8028
MRPS27	MRPS27	mitochondrial ribosomal protein S27	23107	-0.6219
MRPS28	MRPS28	mitochondrial ribosomal protein S28	28957	-1.2515
MRPS30	MRPS30	mitochondrial ribosomal protein S30	10884	-0.6256
MRPS34	MRPS34	mitochondrial ribosomal protein S34	65993	-0.8679
MRPS7	MRPS7	mitochondrial ribosomal protein S7	51081	-1.0273
MRRF	MRRF	mitochondrial ribosome recycling factor	92399	-0.5749
MTHFD1L	MTHFD1L	methylenetetrahydrofolate dehydrogenase (NADP+ dependent) 1 like	25902	-0.579
NAXE	NAXE	NAD(P)HX epimerase	128240	-0.5563
NDUFS1	NDUFS1	NADH:ubiquinone oxidoreductase core subunit S1	4719	-0.7155
NDUFS2	NDUFS2	NADH:ubiquinone oxidoreductase core subunit S2	4720	-0.7935
NDUFS8	NDUFS8	NADH:ubiquinone oxidoreductase core subunit S8	4728	-0.5743
NUDT1	NUDT1	nudix hydrolase 1	4521	-0.5836
OGDH	OGDH	oxoglutarate dehydrogenase	4967	-1.2261
OXA1L	OXA1L	OXA1L, mitochondrial inner membrane protein	5018	-0.5463
PC	PC	pyruvate carboxylase	5091	-0.9681
PDE12	PDE12	phosphodiesterase 12	201626	-0.6229
PDHB	PDHB	pyruvate dehydrogenase E1 beta subunit	5162	-0.9841
PDP2	PDP2	pyruvate dehyrogenase phosphatase catalytic subunit 2	57546	-0.7075
PDSS1	PDSS1	decaprenyl diphosphate synthase subunit 1	23590	-1.2869
PMPCA	PMPCA	peptidase, mitochondrial processing alpha subunit	23203	-1.3778
POLDIP2	POLDIP2	DNA polymerase delta interacting protein 2	26073	-0.7716
POLRMT	POLRMT	RNA polymerase mitochondrial	5442	-1.0525
PRIMPOL	PRIMPOL	primase and DNA directed polymerase	201973	-0.881
PUS1	PUS1	pseudouridine synthase 1	80324	-1.3708
RAD51	RAD51	RAD51 recombinase	5888	-1.1857
RPUSD3	RPUSD3	RNA pseudouridine synthase D3	285367	-1.1347
SARS2	SARS2	seryl-tRNA synthetase 2, mitochondrial	54938	-1.7657
SDHAF1	SDHAF1	succinate dehydrogenase complex assembly factor 1	644096	-0.7909
SDHAF3	SDHAF3	succinate dehydrogenase complex assembly factor 3	57001	-0.6809
SDHAF4	SDHAF4	succinate dehydrogenase complex assembly factor 4	135154	-0.9228
SUCLG2	SUCLG2	succinate-CoA ligase GDP-forming beta subunit	8801	-0.662
TBRG4	TBRG4	transforming growth factor beta regulator 4	9238	-0.7588
TFAM	TFAM	transcription factor A, mitochondrial	7019	-0.5591
TFB1M	TFB1M	transcription factor B1, mitochondrial	51106	-1.3488
TP53	TP53	tumor protein p53	7157	-0.7974
TRAP1	TRAP1	TNF receptor associated protein 1	10131	-0.7914
TRMT10C	TRMT10C	tRNA methyltransferase 10C, mitochondrial RNase P subunit	54931	-0.9892
TRMT5	TRMT5	tRNA methyltransferase 5	57570	-2.3146
TRNT1	TRNT1	tRNA nucleotidyl transferase 1	51095	-0.9303
TRUB2	TRUB2	TruB pseudouridine synthase family member 2	26995	-0.5718

TWNK	TWNK	twinkle mtDNA helicase	56652	-1.0713
TXNRD2	TXNRD2	thioredoxin reductase 2	10587	-1.0878
TYMS	TYMS	thymidylate synthetase	7298	-1.1017
UQCC2	UQCC2	ubiquinol-cytochrome c reductase complex assembly factor 2	84300	-1.0467
WARS2	WARS2	tryptophanyl tRNA synthetase 2, mitochondrial	10352	-0.9778
**ID: GO:0070469; Name: respiratory chain**				
**Size=26; L=25; ES=-0.46; NES=-1.87; PValue=3.937e-3; FDR=1.870e-2**				
**User ID**	**Gene Symbol**	**Gene Name**	**Entrez Gene**	**Score**
BCS1L	BCS1L	BCS1 homolog, ubiquinol-cytochrome c reductase complex chaperone	617	-0.8502
COX1	COX1	cytochrome c oxidase subunit I	4512	-0.3463
COX15	COX15	cytochrome c oxidase assembly homolog COX15	1355	-0.71
COX3	COX3	cytochrome c oxidase III	4514	-0.3833
COX8A	COX8A	cytochrome c oxidase subunit 8A	1351	-0.4121
CYC1	CYC1	cytochrome c1	1537	-0.8942
CYCS	CYCS	cytochrome c, somatic	54205	-0.8343
DMAC1	DMAC1	distal membrane arm assembly complex 1	90871	-0.7157
DMAC2	DMAC2	distal membrane arm assembly complex 2	55101	-0.8339
FOXRED1	FOXRED1	FAD dependent oxidoreductase domain containing 1	55572	-0.8658
NDUFB5	NDUFB5	NADH:ubiquinone oxidoreductase subunit B5	4711	-0.3653
NDUFS1	NDUFS1	NADH:ubiquinone oxidoreductase core subunit S1	4719	-0.7155
NDUFS2	NDUFS2	NADH:ubiquinone oxidoreductase core subunit S2	4720	-0.7935
NDUFS3	NDUFS3	NADH:ubiquinone oxidoreductase core subunit S3	4722	-0.4034
NDUFS8	NDUFS8	NADH:ubiquinone oxidoreductase core subunit S8	4728	-0.5743
NDUFV1	NDUFV1	NADH:ubiquinone oxidoreductase core subunit V1	4723	-0.502
NDUFV3	NDUFV3	NADH:ubiquinone oxidoreductase subunit V3	4731	-1.2657
OXA1L	OXA1L	OXA1L, mitochondrial inner membrane protein	5018	-0.5463
SDHA	SDHA	succinate dehydrogenase complex flavoprotein subunit A	6389	-1.2335
SDHAF4	SDHAF4	succinate dehydrogenase complex assembly factor 4	135154	-0.9228
SDHB	SDHB	succinate dehydrogenase complex iron sulfur subunit B	6390	-0.3149
TTC19	TTC19	tetratricopeptide repeat domain 19	54902	-0.6218
UQCRC1	UQCRC1	ubiquinol-cytochrome c reductase core protein 1	7384	-0.4335
UQCRC2	UQCRC2	ubiquinol-cytochrome c reductase core protein 2	7385	-0.6507
UQCRFS1	UQCRFS1	ubiquinol-cytochrome c reductase, Rieske iron-sulfur polypeptide 1	7386	-0.3548
**ID: GO:0098798; Name: mitochondrial protein complex**				
**Size=118; L=110; ES=-0.43; NES=-2.51; PValue=0.000e+0; FDR=0.000e+0**				
**User ID**	**Gene Symbol**	**Gene Name**	**Entrez Gene**	**Score**
AFG3L2	AFG3L2	AFG3 like matrix AAA peptidase subunit 2	10939	-0.4887
AGK	AGK	acylglycerol kinase	55750	-0.3997
ATP5F1A	ATP5F1A	ATP synthase F1 subunit alpha	498	-0.4815
ATP5F1B	ATP5F1B	ATP synthase F1 subunit beta	506	-0.3472
ATP5F1D	ATP5F1D	ATP synthase F1 subunit delta	513	-0.7817
ATP5MC1	ATP5MC1	ATP synthase membrane subunit c locus 1	516	-0.8153
ATP5MC3	ATP5MC3	ATP synthase membrane subunit c locus 3	518	-0.4612
ATP5PB	ATP5PB	ATP synthase peripheral stalk-membrane subunit b	515	-0.3043
BAX	BAX	BCL2 associated X, apoptosis regulator	581	-0.8407
BCKDHB	BCKDHB	branched chain keto acid dehydrogenase E1 subunit beta	594	-0.8234
BCKDK	BCKDK	branched chain ketoacid dehydrogenase kinase	10295	-1.1649
BCS1L	BCS1L	BCS1 homolog, ubiquinol-cytochrome c reductase complex chaperone	617	-0.8502
CHCHD6	CHCHD6	coiled-coil-helix-coiled-coil-helix domain containing 6	84303	-0.5252
COX1	COX1	cytochrome c oxidase subunit I	4512	-0.3463
COX3	COX3	cytochrome c oxidase III	4514	-0.3833
CYC1	CYC1	cytochrome c1	1537	-0.8942
DAP3	DAP3	death associated protein 3	7818	-0.2985
DLAT	DLAT	dihydrolipoamide S-acetyltransferase	1737	-0.3558
DMAC1	DMAC1	distal membrane arm assembly complex 1	90871	-0.7157
DMAC2	DMAC2	distal membrane arm assembly complex 2	55101	-0.8339
DNA2	DNA2	DNA replication helicase/nuclease 2	1763	-1.2112
DNAJC11	DNAJC11	DnaJ heat shock protein family (Hsp40) member C11	55735	-0.9315
FOXRED1	FOXRED1	FAD dependent oxidoreductase domain containing 1	55572	-0.8658
GRPEL1	GRPEL1	GrpE like 1, mitochondrial	80273	-0.5003
HSD17B10	HSD17B10	hydroxysteroid 17-beta dehydrogenase 10	3028	-0.3435
IMMT	IMMT	inner membrane mitochondrial protein	10989	-0.4827
MCCC2	MCCC2	methylcrotonoyl-CoA carboxylase 2	64087	-0.3145

MCU	MCU	mitochondrial calcium uniporter	90550	-0.6009
MICU2	MICU2	mitochondrial calcium uptake 2	221154	-0.8139
MRPL11	MRPL11	mitochondrial ribosomal protein L11	65003	-0.4185
MRPL12	MRPL12	mitochondrial ribosomal protein L12	6182	-1.8151
MRPL14	MRPL14	mitochondrial ribosomal protein L14	64928	-0.4118
MRPL15	MRPL15	mitochondrial ribosomal protein L15	29088	-0.9279
MRPL16	MRPL16	mitochondrial ribosomal protein L16	54948	-0.7676
MRPL17	MRPL17	mitochondrial ribosomal protein L17	63875	-1.2273
MRPL19	MRPL19	mitochondrial ribosomal protein L19	9801	-0.6191
MRPL2	MRPL2	mitochondrial ribosomal protein L2	51069	-0.6308
MRPL20	MRPL20	mitochondrial ribosomal protein L20	55052	-0.9108
MRPL21	MRPL21	mitochondrial ribosomal protein L21	219927	-0.7527
MRPL24	MRPL24	mitochondrial ribosomal protein L24	79590	-0.7585
MRPL27	MRPL27	mitochondrial ribosomal protein L27	51264	-1.0032
MRPL28	MRPL28	mitochondrial ribosomal protein L28	10573	-0.9266
MRPL3	MRPL3	mitochondrial ribosomal protein L3	11222	-0.5017
MRPL32	MRPL32	mitochondrial ribosomal protein L32	64983	-0.65
MRPL35	MRPL35	mitochondrial ribosomal protein L35	51318	-1.0054
MRPL36	MRPL36	mitochondrial ribosomal protein L36	64979	-0.9912
MRPL37	MRPL37	mitochondrial ribosomal protein L37	51253	-0.5457
MRPL4	MRPL4	mitochondrial ribosomal protein L4	51073	-0.6947
MRPL42	MRPL42	mitochondrial ribosomal protein L42	28977	-0.5884
MRPL43	MRPL43	mitochondrial ribosomal protein L43	84545	-0.3745
MRPL49	MRPL49	mitochondrial ribosomal protein L49	740	-0.4066
MRPL50	MRPL50	mitochondrial ribosomal protein L50	54534	-0.7727
MRPL52	MRPL52	mitochondrial ribosomal protein L52	122704	-0.8534
MRPL54	MRPL54	mitochondrial ribosomal protein L54	116541	-0.8803
MRPL55	MRPL55	mitochondrial ribosomal protein L55	128308	-0.5606
MRPL57	MRPL57	mitochondrial ribosomal protein L57	78988	-1.1413
MRPL58	MRPL58	mitochondrial ribosomal protein L58	3396	-1.0237
MRPL9	MRPL9	mitochondrial ribosomal protein L9	65005	-0.4901
MRPS10	MRPS10	mitochondrial ribosomal protein S10	55173	-0.3615
MRPS11	MRPS11	mitochondrial ribosomal protein S11	64963	-0.4568
MRPS12	MRPS12	mitochondrial ribosomal protein S12	6183	-1.0623
MRPS14	MRPS14	mitochondrial ribosomal protein S14	63931	-0.8098
MRPS15	MRPS15	mitochondrial ribosomal protein S15	64960	-0.3156
MRPS16	MRPS16	mitochondrial ribosomal protein S16	51021	-0.5475
MRPS17	MRPS17	mitochondrial ribosomal protein S17	51373	-1.2133
MRPS18B	MRPS18B	mitochondrial ribosomal protein S18B	28973	-0.7077
MRPS2	MRPS2	mitochondrial ribosomal protein S2	51116	-0.7504
MRPS26	MRPS26	mitochondrial ribosomal protein S26	64949	-0.8028
MRPS27	MRPS27	mitochondrial ribosomal protein S27	23107	-0.6219
MRPS28	MRPS28	mitochondrial ribosomal protein S28	28957	-1.2515
MRPS30	MRPS30	mitochondrial ribosomal protein S30	10884	-0.6256
MRPS33	MRPS33	mitochondrial ribosomal protein S33	51650	-0.4104
MRPS34	MRPS34	mitochondrial ribosomal protein S34	65993	-0.8679
MRPS35	MRPS35	mitochondrial ribosomal protein S35	60488	-0.4987
MRPS7	MRPS7	mitochondrial ribosomal protein S7	51081	-1.0273
MRPS9	MRPS9	mitochondrial ribosomal protein S9	64965	-0.4876
MTERF4	MTERF4	mitochondrial transcription termination factor 4	130916	-0.5296
MTX1	MTX1	metaxin 1	4580	-0.9412
NDUFB5	NDUFB5	NADH:ubiquinone oxidoreductase subunit B5	4711	-0.3653
NDUFS1	NDUFS1	NADH:ubiquinone oxidoreductase core subunit S1	4719	-0.7155
NDUFS2	NDUFS2	NADH:ubiquinone oxidoreductase core subunit S2	4720	-0.7935
NDUFS3	NDUFS3	NADH:ubiquinone oxidoreductase core subunit S3	4722	-0.4034
NDUFS8	NDUFS8	NADH:ubiquinone oxidoreductase core subunit S8	4728	-0.5743
NDUFV1	NDUFV1	NADH:ubiquinone oxidoreductase core subunit V1	4723	-0.502
NDUFV3	NDUFV3	NADH:ubiquinone oxidoreductase subunit V3	4731	-1.2657
PNPT1	PNPT1	polyribonucleotide nucleotidyltransferase 1	87178	-1.3887
POLRMT	POLRMT	RNA polymerase mitochondrial	5442	-1.0525
SAMM50	SAMM50	SAMM50 sorting and assembly machinery component	25813	-0.4162
SDHA	SDHA	succinate dehydrogenase complex flavoprotein subunit A	6389	-1.2335
SDHAF4	SDHAF4	succinate dehydrogenase complex assembly factor 4	135154	-0.9228
SDHB	SDHB	succinate dehydrogenase complex iron sulfur subunit B	6390	-0.3149
SPG7	SPG7	SPG7, paraplegin matrix AAA peptidase subunit	6687	-0.709
SUCLG2	SUCLG2	succinate-CoA ligase GDP-forming beta subunit	8801	-0.662
TIMM10B	TIMM10B	translocase of inner mitochondrial membrane 10B	26515	-1.1482

TIMM13	TIMM13	translocase of inner mitochondrial membrane 13	26517	-0.8215
TIMM17A	TIMM17A	translocase of inner mitochondrial membrane 17A	10440	-0.6497
TIMM21	TIMM21	translocase of inner mitochondrial membrane 21	29090	-0.5245
TIMM22	TIMM22	translocase of inner mitochondrial membrane 22	29928	-0.6646
TIMM50	TIMM50	translocase of inner mitochondrial membrane 50	92609	-0.5457
TIMM8B	TIMM8B	translocase of inner mitochondrial membrane 8 homolog B	26521	-0.6737
TOMM20	TOMM20	translocase of outer mitochondrial membrane 20	9804	-0.4694
TOMM22	TOMM22	translocase of outer mitochondrial membrane 22	56993	-0.8073
TOMM40	TOMM40	translocase of outer mitochondrial membrane 40	10452	-1.6586
TOMM40L	TOMM40L	translocase of outer mitochondrial membrane 40 like	84134	-1.6432
TOMM5	TOMM5	translocase of outer mitochondrial membrane 5	401505	-1.3801
TRMT10C	TRMT10C	tRNA methyltransferase 10C, mitochondrial RNase P subunit	54931	-0.9892
UQCRC1	UQCRC1	ubiquinol-cytochrome c reductase core protein 1	7384	-0.4335
UQCRC2	UQCRC2	ubiquinol-cytochrome c reductase core protein 2	7385	-0.6507
UQCRFS1	UQCRFS1	ubiquinol-cytochrome c reductase, Rieske iron-sulfur polypeptide 1	7386	-0.3548
VDAC1	VDAC1	voltage dependent anion channel 1	7416	-0.3212
