# Supplementary material for: Autophagy mitigates ethanol-induced mitochondrial dysfunction and oxidative stress in esophageal keratinocytes
Source: PLoS One. 2020 Sep 23;15(9):e0239625. doi: 10.1371/journal.pone.0239625 (PMC7510980; doi:10.1371/journal.pone.0239625)
Supplement: S7 Fig — (PDF) [file pone.0239625.s007.pdf]

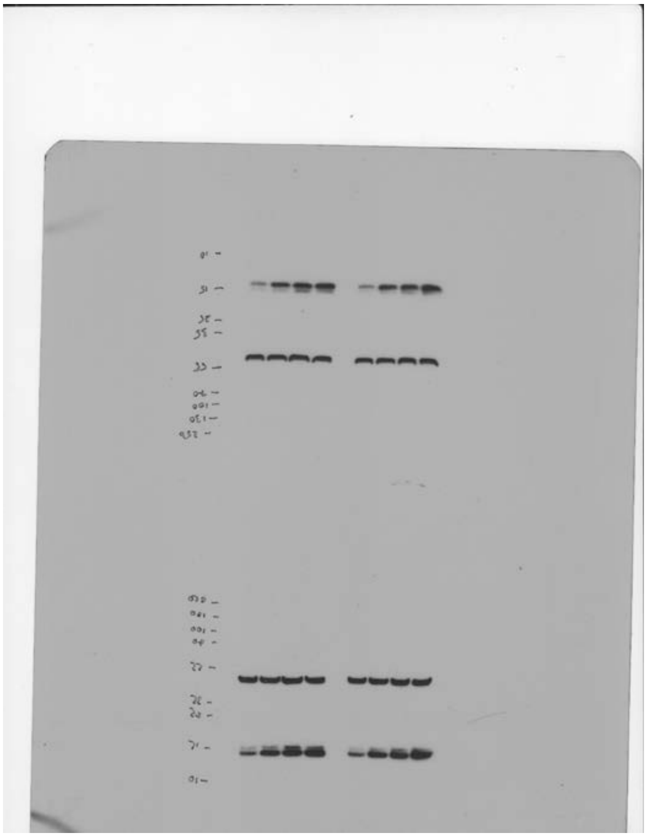

LC3B and b-actin (EPC1/EPC2)

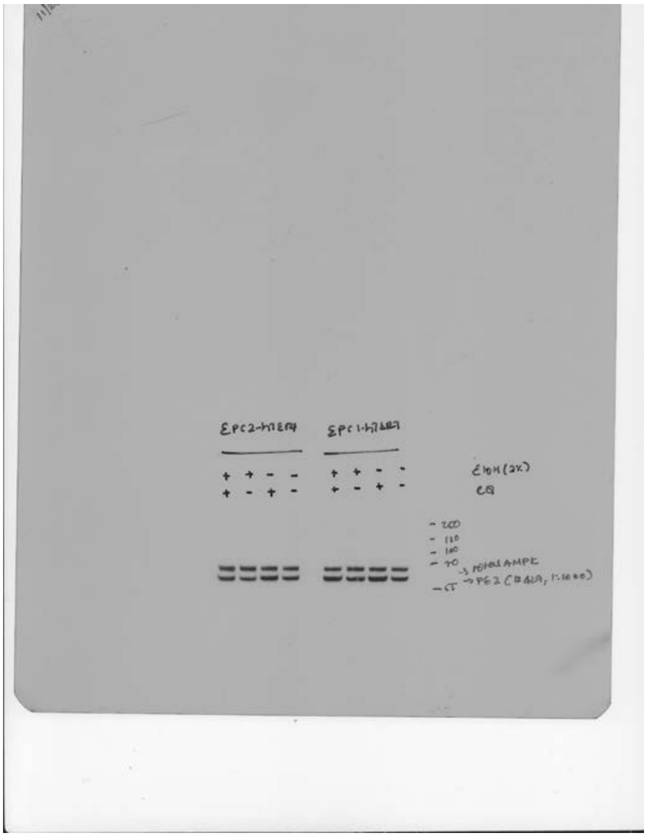

P62 (and AMPK) (EPC1/EPC2)

S7 Fig. Original, uncropped and minimally adjusted images of immunoblots shown in Fig. 7A
